# A Randomized Control Study to Assess the Efficacy of Intrathecal Morphine in Patients on Patient-Controlled Analgesia Pump With Morphine for Postoperative Pain Relief After Elective Laparotomy

**DOI:** 10.7759/cureus.52741

**Published:** 2024-01-22

**Authors:** Anand Kuppusamy, Sujina Hermin Angel, Karthik Kandan, Balasubramaniam Gayathri

**Affiliations:** 1 Anaesthesiology, SRM Medical College Hospital and Research Centre, Chennai, IND

**Keywords:** analgesics, enhanced recovery, morphine, pruritis, postoperative nausea and vomiting, laparotomy, intrathecal injection

## Abstract

Introduction

Laparotomy is associated with significant prolonged postoperative pain, which can cause an adverse neuroendocrine stress response. Intrathecal morphine (ITM) retains an important place in pain management after major laparotomy since it is easier to administer and has a relatively lesser possibility of failure and technical difficulty.

Aim

The study aims to compare the effect of the administration of ITM with intravenous (IV) morphine administered by a patient-controlled analgesia (PCA) pump on postoperative analgesia after elective laparotomy. The primary objective was to compare total morphine consumption while secondary objectives were to compare pain assessed by the visual analog scale (VAS) and adverse reactions to opioids.

Methods

Sixty patients who underwent elective laparotomy were enrolled in this study. Thirty patients were enrolled in the study group (ITM+PCA) where ITM (200 mcg) was administered before laparotomy and intravenous morphine was initiated with PCA postoperatively. In the control group, only intravenous morphine was given with PCA postoperatively for pain relief. Parameters in both groups were compared, where estimation of cumulative morphine dose was the primary outcome and pain as assessed by VAS and side effects of opioids were the secondary outcomes.

Results

Patients in the ITM (ITM+PCA) group required less morphine (6.6 ± 2.96 vs. 24.77 ± 6.79 mg of morphine, p < 0.001) compared to patients on PCA. There was no statistically significant difference in VAS score and adverse effects between both groups.

Conclusion

Preoperative ITM can be used as an effective and safe modality for alleviating immediate postoperative pain following laparotomy.

## Introduction

The International Association for the Study of Pain and the World Health Organization both regard access to pain relief as a fundamental human right [1]. Laparotomy is associated with significant, prolonged postoperative pain. Poor acute pain control in postoperative patients has a variety of detrimental effects, such as accelerated morbidity, decreased quality of life and physical function, and prolonged recovery [2]. A reasonable foundation for improved postoperative pain control, analgesia optimization, a reduction in side effects, and increased patient satisfaction is provided by the employment of a procedure-specific, multimodal perioperative pain management strategy [3].

Various modalities of postoperative analgesia after laparotomy include paracetamol, non-steroidal anti-inflammatory drugs (NSAIDs), intravenous opioids or epidural local anesthetics administered by a patient-controlled analgesia (PCA) pump, and truncal blocks [4]. Paracetamol and NSAIDs are used as part of multimodal analgesia but are ineffective as sole analgesics following laparotomy. Intravenous opioids administered by a PCA pump provide good analgesia but can cause nausea, vomiting, pruritis, and respiratory depression [5]. Epidural anesthesia with local anesthetics and opioids provides good analgesia but can cause hypotension, urinary retention, pruritis, and delayed ambulation due to motor block. Truncal blocks spare visceral pain but can be administered as a single injection only and require ultrasound for precise performance.

Enhanced Recovery After Surgery (ERAS) is a scientific methodology used to improve postoperative recovery and outcomes [6]. Adequate postoperative analgesia after major surgery is one of the important prerequisites for ERAS. Intrathecal hydrophilic opioids with high potency and extended duration of action without motor block are ideal agents to achieve the objectives of ERAS. Other advantages of intrathecal hydrophilic opioids include single-dose administration, the absence of motor blockade, which enables early ambulation, and the absence of vasodilatation, which enables restrictive fluid management [7]. On perusal of the literature, there is insufficient evidence to assess the effect of the administration of intrathecal morphine (ITM) with intravenous morphine in the PCA pump on postoperative analgesia after elective laparotomy. Hence, we devised this study to investigate the effects of ITM for postoperative analgesia in laparotomy.

## Materials and methods

Study population

The randomized, single-blinded study was conducted with patients posted for major abdominal surgeries at a tertiary teaching hospital in India after obtaining institutional ethics committee approval (IEC No: 1155/IEC/2017) and registration in the Clinical Trial Registry of India (CTRI/2018/08/015183). After obtaining informed written consent from all study participants, the study was conducted in adherence to the Helsinki Declaration. We enrolled 63 patients in this study. Three patients refused to participate in the trial due to apprehensions about spinal anesthesia. Hence, 60 patients assessed under the American Society of Anesthesiologists physical status I-II, ages 18-65 years, undergoing elective laparotomy, participated in this study and were randomly allotted to either the ITM+PCA or PCA groups. Patients who had contraindications for subarachnoid block, who were not able to use PCA pumps due to psychiatric illness, or who had a history of opioid abuse or an allergy to opioids were excluded from the study. All the patients were taught to use the PCA and to evaluate pain using the visual analog scale (VAS) on the day before the procedure.

Randomization and anesthetic management

A computer-generated random number table was used to assign patients to one of the two groups at random. After opening the group assignment envelope, an anesthesiology resident who was not involved in the anesthetic induction prepared a syringe with 200 mcg of morphine diluted to 1 ml. A consultant anesthesiologist performed spinal anesthesia and collected the data. Patients in the ITM+PCA group received 200 mcg of ITM in a sitting position at L3-L4 lumbar vertebral level with a 25G Quincke spinal needle, while those in the PCA group were not administered any intrathecal drug due to ethical reasons. Induction of anesthesia was done with injection (Inj.) propofol 2 mg/kg and Inj. fentanyl 2 mcg/kg, and the trachea was intubated after administering Inj. vecuronium 0.1 mg/kg. A mixture of air and oxygen (50:50), 1%-2% sevoflurane, and a bolus of 1 mcg/kg/hour fentanyl were used to maintain anesthesia. At the end of the surgery, the neuromuscular blockade was reversed with Inj. neostigmine 0.05 mg/kg and extubated after ensuring adequate recovery. Patients were initially administered a 0.05 mg/kg bolus of morphine by PCA pump without base flow if their pain score was higher than 6 on a 10 cm VAS scale in the recovery room. In the post-anesthesia care unit (PACU), the pump was reprogrammed to deliver 1 mg morphine without basal flow, with a lockout time of 10 minutes. PCA was maintained during the initial 48 hours postoperatively. All patients in both groups received intravenous paracetamol (1 g) thrice daily for an initial 72 hours. Intravenous ondansetron was given as a rescue medication to manage nausea and vomiting. Patients were administered Inj. fentanyl 1 mcg/kg intravenously if VAS > 4 despite intravenous paracetamol and morphine PCA. Oxygen saturation and respiratory rate were monitored to diagnose respiratory depression. When peripheral oxygen saturation was less than 90% as detected by a pulse oximeter or the respiratory rate was less than 8/minute, the patients were diagnosed with respiratory depression and were administered supplemental oxygen.

Outcome evaluation

The healthcare personnel who collected data postoperatively were blinded to the study group. The intensity of pain was assessed using a VAS. At one, six, 12, 18, 24, 36, and 48 hours following surgery, patients completed pain questionnaires with scores ranging from 0 mm for total pain relief to 100 mm for the most excruciating pain imaginable. Incidences of postoperative nausea, vomiting, and pruritis were noted.

Statistical analysis

In the pilot study, the difference in VAS score between the ITM group and the IV PCA morphine group was 2.1. It was determined that a sample size of 60 patients with 30 in each group would provide 90% statistical power and 0.05 alpha error. Every result was presented as a mean with a standard deviation, median (range), or number (percentage). Continuous variables were analyzed using the unpaired t-test. The chi-square test was employed to analyze categorical variables. The cutoff for statistical significance was p <= 0.05. Data were entered in Microsoft Excel 2020 (Microsoft Corporation, Redmond, WA) and SPSS version 16 (SPSS Inc., Chicago, IL) was used to analyze the same.

## Results

**Figure 1:**
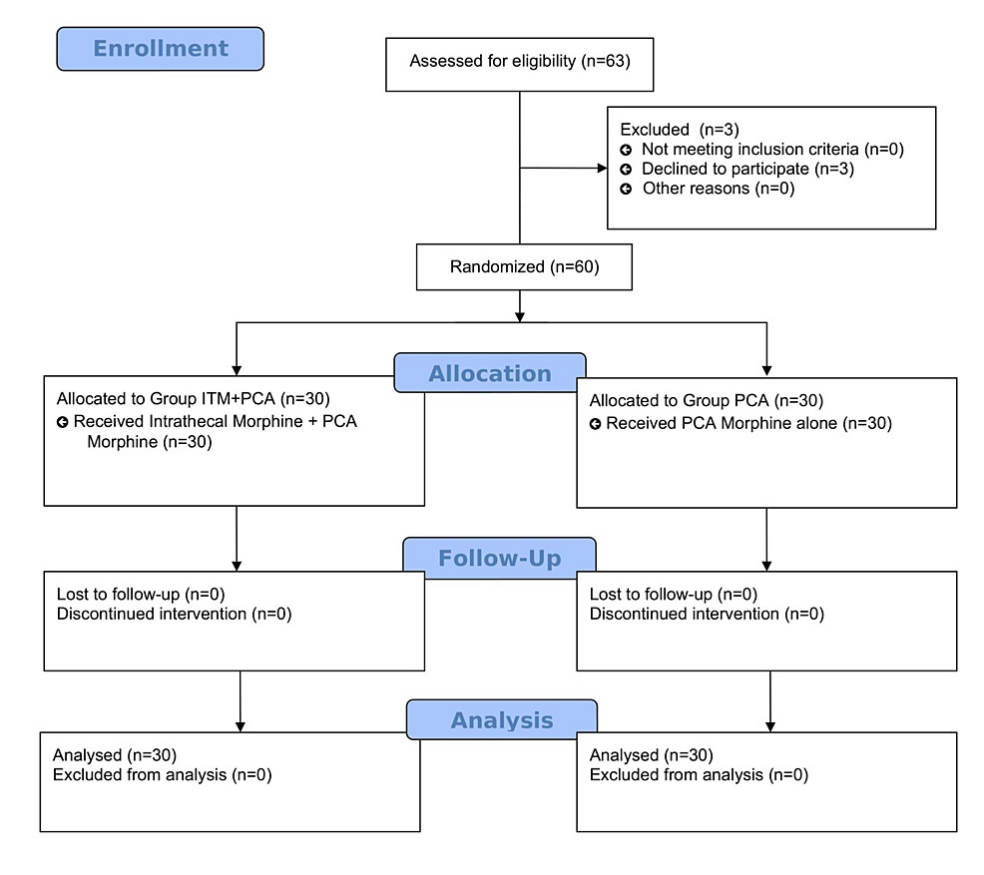
CONSORT flow diagram CONSORT: Consolidated Standards of Reporting Trials; ITM: intrathecal morphine; PCA: patient-controlled analgesia.

Patient flow in the trial as per CONSORT (Consolidated Standards of Reporting Trials) 2010 is shown in Figure 1.

Patient characteristics

Patients in both groups were statistically equivalent with respect to demographic variables like age, gender, weight, and the American Society of Anesthesiologists (ASA) physical status (Table 1).

**Table 1:** Comparison of patients’ characteristics Values are presented in mean ± SD or number of patients. Age and weight were compared by t-test. Group PCA: Patient-controlled analgesia without intrathecal morphine. Group ITM+PCA: Patient-controlled analgesia with intrathecal morphine. ASA PS: American Society of Anesthesiologists Physical Status.

Factors		Group ITM+PCA	Group PCA	P-value
Age		47.70 ± 8.67	43.07 ± 12.39	0.099
Gender	Male	13 (43%)	12 (40%)	
	Female	17 (57%)	18 (60%)	
Weight		58.50 ± 7.58	60.40 ± 7.67	0.339
ASA PS	I	21 (70%)	24 (80%)	
	II	9 (30%)	6 (20%)	

Total morphine consumption

**Figure 2:**
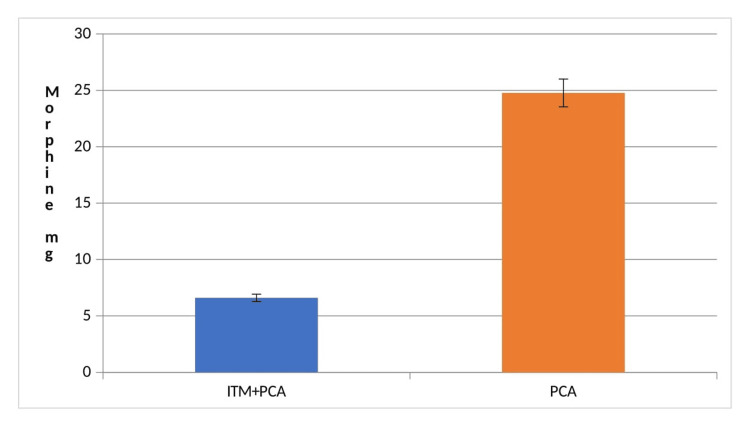
Postoperative morphine consumption Group PCA: Patient-controlled analgesia without intrathecal morphine. Group ITM+PCA: Patient-controlled analgesia with intrathecal morphine.

The patients who received ITM in the ITM+PCA group consumed 6.6 ± 2.96 mg of morphine, while patients who used PCA alone consumed 24.77 ± 6.79 mg of morphine in the first 48 hours. The difference between the groups was statistically significant (p < 0.001). None of the patients in the ITM+PCA group required rescue analgesia, while only three patients in the PCA group required the same (Figure 2).

VAS scores

**Figure 3:**
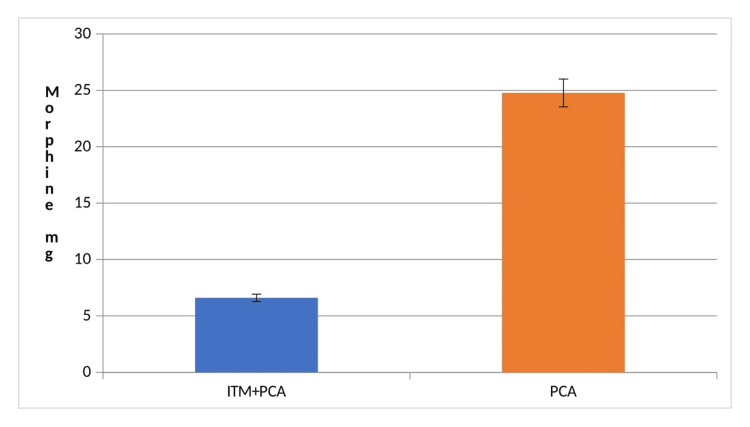
Visual analog scale (VAS) score Group PCA: Patient-controlled analgesia without intrathecal morphine. Group ITM+PCA: Patient-controlled analgesia with intrathecal morphine.

The analysis of VAS in both groups up to 48 hours revealed no statistically significant difference between the two groups, with a p-value > 0.05 (Figure 3).

Adverse effects

The Ramsay sedation score was measured at one, six, 12, 18, 24, 36, and 48 hours. Patients who were administered ITM (group ITM+PCA) were sedated more significantly at one, six, 12, 18, and 24 hours, with p < 0.05, compared to those in the PCA group. Twenty-eight patients in the ITM+PCA group and 17 patients in the PCA-only group had nausea, but the difference was not statistically significant. Though 90% of patients in the ITM+PCA group had pruritis, compared to 70% of patients in the PCA group, the difference was not statistically significant, with a p-value of 0.104. None of the patients in either group developed respiratory depression in the first 48 hours (Table 2).

**Table 2:** Adverse effects of intrathecal morphine Values are presented as mean ± SD or number of patients. Group PCA: Patient-controlled analgesia without intrathecal morphine. Group ITM+PCA: Patient-controlled analgesia with intrathecal morphine. * Respiratory depression = respiratory rate < 8/minute or oxygen saturation (SpO2) < 90%.

Parameters			Group ITM+PCA	Group PCA	P-value
Visual analog scale (VAS)	1 hour		12.33 ± 6.26	15.33 ± 5.71	0.57
	6 hours		10.33 ± 5.24	12.67 ± 5.04	0.84
	12 hours		9.33 ± 3.65	11 ± 3.05	0.06
	18 hours		8.5 ± 2.33	9.33 ± 1.72	0.121
	24 hours		9.83 ± 4.04	11 ± 4.43	0.291
	36 hours		7.83 ± 2.52	8.17 ± 2.45	0.605
	48 hours		7 ± 2.49	7.17 ± 2.52	0.798
Total morphine consumption (mg)			6.6 ± 2.96	24.77 ± 6.79	0.0000
Ramsay sedation score (hours)		1	2.12 ± 0.24	1.8 ± 0.55	0.01
		6	2.63 ± 0.49	2.27 ± 0.52	0.007
		12	2.73 ± 0.34	2.37 ± 0.45	0.04
		18	2.83 ± 0.34	2.4 ± 0.49	0.02
		24	2.57 ± 0.25	2.3 ± 0.46	0.02
		36	2.1 ± 0.3	2 ± 0	0.078
		48	2 ± 0	2 ± 0	1
Nausea			28 (93.3%)	17 (56.7%)	0.002
Pruritis			27 (90%)	21 (70%)	0.104
Respiratory depression^*^			0	0	NS

## Discussion

Our study aimed to investigate the challenges in the integration of ITM within the ambit of ERAS protocols for abdominal procedures. The fundamental objective of ERAS in curbing surgical stress responses by optimizing postoperative care includes the crucial aspect of optimal pain management [4]. In this context, ITM stands out as a potential option to reduce systemic opioids. Our findings highlight a complex interplay of benefits and challenges associated with the usage of ITM in abdominal surgeries.

Our study affirmed the potency of ITM in reducing postoperative morphine requirements among patients utilizing PCA. Consistent with existing literature, this underscores the opioid-sparing potential of ITM, although variations in dosage and patient demographics among studies suggest the need for more detailed research [4]. Factors such as genetic variations, surgical procedures, and individual patient characteristics seemingly contribute to the divergent outcomes in morphine requirements observed across studies employing ITM. ITM has been proven to reduce analgesic requirements in a variety of surgical procedures like obstetrics, spinal surgeries, arthroplasty, urology, and thoracic surgeries [8-12]. There is no proof of linear dose-responsiveness for any of the advantageous or detrimental effects across a wide range of dosages. Currently, depending on the type of surgery, intrathecal doses vary from 0.1 to 0.5 mg while relatively large doses as high as 4 mg were utilized in the late 20th century [7].

However, the vital concern regarding the balance between analgesic efficacy and the incidence of adverse effects warrants attention. While pain scores, evaluated through the VAS, exhibited similarity between ITM+PCA and PCA-only groups over 48 hours, the former showcased a diminished demand for morphine to achieve these scores. This emphasizes ITM's role in potentially achieving comparable pain relief with reduced opioid consumption, a crucial aspect in the quest for opioid minimization strategies within ERAS.

The incidence of adverse effects, particularly nausea, vomiting, and sedation, in the ITM+PCA cohort necessitates careful consideration. The prevalence of nausea in ITM recipients is similar to previous studies, indicating a substantial concern in its clinical application [13-16]. None of our patients developed respiratory depression as defined by oxygen saturation (SpO2) < 90% till 48 hours postoperatively. However, there was no standard definition for respiratory depression in published literature. Respiratory rate of less than eight or even 10 breaths per minute (bpm), less than 85% or even 96% oxygen saturation, or the requirement for naloxone to maintain a sufficient tidal volume are some examples of definitions for respiratory depression in previously published research articles [7].

This emphasizes the necessity to strike a balance between adequate pain control and reducing adverse effects. Dose optimization and supplementary measures might hold promise in mitigating these complications while leveraging the analgesic benefits of ITM.

In conclusion, our study underscores the potential of ITM as a means to reduce postoperative opioid consumption without compromising pain relief within ERAS protocols for abdominal procedures. However, careful consideration is warranted concerning the risk-benefit ratio, particularly regarding adverse effects. Optimizing dosages and exploring adjunctive therapies may lead to maximizing the analgesic advantages of ITM while reducing associated complications, fostering its judicious integration into ERAS protocols for improved postoperative outcomes. Further studies focusing on refining standardized protocols and evaluating additional measures are imperative to delineate the optimal role of ITM in ERAS protocol.

Limitations

Though multiple doses of ITM are used in literature, we finalized 200 mcg based on our institutional protocol, which produces optimal analgesia with the least side effects. We investigated postoperative analgesic requirements in patients who were given ITM, while the quality of recovery (QoR) would have been a better parameter to assess the postoperative well-being of patients.

## Conclusions

A single spinal injection of morphine combined with an intravenous PCA pump with morphine provided more effective postoperative analgesia than an intravenous PCA pump with morphine alone and reduced opioid requirements in patients undergoing elective laparotomy procedures. These results suggest that preoperative ITM can be used as an effective and safe modality for alleviating immediate postoperative pain following laparotomy.
